# Effects of Antarctic krill oil on lipid and glucose metabolism in C57BL/6J mice fed with high fat diet

**DOI:** 10.1186/s12944-017-0601-8

**Published:** 2017-11-21

**Authors:** Dewei Sun, Liang Zhang, Hongjian Chen, Rong Feng, Peirang Cao, Yuanfa Liu

**Affiliations:** State Key Laboratory of Food Science and Technology, Synergetic Innovation Center of Food Safety and Nutrition, School of Food Science and Technology, Jiangnan University, 1800 Lihu Avenue, Wuxi, Jiangsu 214122 People’s Republic of China

**Keywords:** Antarctic Krill oil, EPA and DHA, C57BL/6J mice, Lipid metabolism, Glucose tolerance

## Abstract

**Background:**

Obesity and other metabolic diseases have become epidemic which greatly affect human health. Diets with healthy nutrition are efficient means to prevent this epidemic occurrence. Novel food resources and process technology were needed for these purpose. In this study, Antarctic krill oil (KO) extracted from a dry krill by a procedure of hot pump dehydration in combined with freezing-drying was used to investigate health effect in animals including the growth, lipid and glucose metabolism.

**Methods:**

C57BL/6J mice were fed with a lard based high fat (HF) diet and substituted with KO for a period of 12 weeks in comparison with low fat normal control (NC) diet. Mice body weight and food consumption were recorded. Serum lipid metabolism - of C57BL/6J mice serum was measured. A glucose tolerance tests (GTTs) and pathology analysis of mice were performed at the end of the experiment.

**Results:**

The KO fed mice had less body weight gain, less fat accumulation in tissue such as adipose and liver. Dyslipidemia induced by high fat diet was partially improved by KO feeding with significant reduction of serum low density lipoprotein-cholesterol (LDL-C) content. Furthermore, KO feeding also improved glucose metabolism in C57BL/6J mice including a glucose tolerance of about 22% vs. 32% of AUC (area under the curve) for KO vs HF diet and the fast blood glucose level of 8.5 mmol/L, 9.8 mmol/L and 9.3 mmol/L for NC, HF and KO diet groups, respectively. In addition, KO feeding also reduced oxidative damage in liver with a decrease of malondialdehyde (MDA) content and increase of superoxide dismutase (SOD) content.

**Conclusion:**

This study provided evidence of the beneficial effects of KO on animal health from the processed technology, particularly on lipid and glucose metabolism. This study confirmed that as the Antarctic krill was extracted with a procedure of efficient energy, it might make it possible for Krill oil to be available for food industry.

## Background

Obesity and diabetes become epidemic and a major burden for public health in the developed countries and in the world. Individuals with obesity are at a higher risk of chronic diseases including cardiovascular diseases, nonalcoholic fatty liver disease, type 2 diabetes [1] and other metabolic syndromes.

Obesity is associated with intrahepatic lipid accumulation, which has been linked to the development of insulin resistance and metabolic dysfunction, eventually to an occurrence of diabetes. Lifestyle factors such as diet and physical activity influence obesity through change of adiposity and insulin resistance. Diets with proper food ingredients have great impact on the health of population, such as supplementation with nutraceuticals. Unsaturated fatty acid supplementation, particularly of marine-derived oils from fish, seaweed, microalgae and Antarctic krill, have been popular for their high contents of fatty acid docosahexaenoic acid (DHA, 22:6 ω3) and eicosapentaenoic acid (EPA, 20:5 ω3). These n-3 fatty acids have been documented for their protective effects on cardiovascular diseases, diabetes and chronic metabolic diseases [2].

Krill is an increasingly important source of n-3 PUFAs as Krill oil has high EPA and DHA-containing phospholipids which might be better bioavailability with some uncertainty [3]. KO has a significant amount of astaxanthin, an important natural antioxidative component [4]. Intake of food rich in antioxidants is beneficial to reduce the risk of the cardiovascular, high blood pressure and cancer [5]. KO in animal feeding with high fat diet has been demonstrated to improve its dyslipidemia, body weight and glucose metabolism [6–8]. KO containing diets can significantly improve fast blood glucose concentration and glucose intolerance leading to enhance insulin sensitivities in obese animals [8–10].

KO as a food supplementation has become popular with some pilot trials indicating healthy benefits [11]. Beuy et al., had reviewed the biological function of the KO, and pointed out that KO seems to be good marine food supplementation but there is still no concrete conclusive on clinical efficacy in the management of chronic metabolic diseases [12].

As Antarctic krill is a huge bio-resource of value food oil with less polluted, proper technology development is needed for exploration as nutrient supplementation for food industry. This lab had developed a novel preservation procedure for Antarctic krill with energy efficiency and could be applied in a large scale for food industry [13]. Since the different technologies could greatly impact oil properties and healthy effect as well, in this study, the aim was to investigate the effects of KO, extracted from Antarctic krill using a drying procedure with energy efficiency on C57BL/6J mice with experimentally induced obesity. The mice fed with high fat diet, substituent of KO in contrast to normal chow diet showed that KO diet could reduce animal body weight gain and improve dyslipidemia, glucose metabolism as well as oxidative damage.

## Methods

### KO preparation and reagents

Frozen Antarctic krill were provided by Liaoning Province Dalian Ocean Fishery Group of Corporations (Dalian, China) and dried by hot pump dehydration system (HGOE-10/s, Hangzhou Ouyi Electric Co., Ltd., Hangzhou, China) combined with freezing-drying procedure [13]. KO was extracted by a subcritical extraction system (CBE-5 L, Henan Yalinjie Biological Technology Co., Ltd., Anyang, China) using butane as subcritical fluid, then stored at −40 °C for further use.

Fatty acid methyl ester standards were purchased from Sigma-Aldrich (St. Louis, USA). Hematoxylin and eosin were obtained from Nanjing Jiancheng Bioengineering Institute (Nanjing, China). All other reagents were analytical grade and purchased from Sinopharm Chemical Reagent Co., Ltd. (Shanghai, China).

### KO profile analysis

Peroxide Value (POV) was performed according to the American Oil Chemists’ Society (AOCS) Method Cd 8–53 [14]. Fatty acids analysis was evaluated by fatty acid derivatives of methyl ester, and detected by Gas chromatography according to the method of AOCS [15]. PLs were measured by an HPLC system (Waters 600) equipped with a UV detector (Waters-2487, USA) according to the method by Jiang et al. [16]. Tocopherol content was determined and quantified by using an HPLC system (LC-20AT, Shimadzu, Japan) according to AOCS Method Ce 8–89 [17], and the contents were reported in mg/kg.

Astaxanthin content was measured by using a UV spectrophotometer (Alpha-1500, Shanghai Puyuan instrument Co. Ltd., Shanghai, China) according to the method of Tolasa and Brown et al. [18, 19]. Astaxanthin concentration was calculated from the standard curve of astaxanthin [18].

### Animals and diets

In this study, 30 male C57BL/6J mice (SLRC Laboratory Animal, Shanghai, China) at 6 weeks of age were randomly divided into 3 groups after being fed with normal rodent chow (SLRC Laboratory Animal, Shanghai, China) for a week for acclimation. Mice were fed with following diets: normal low-fat chow diet, lard based high fat diet and KO containing high fat diet as indicated in Table 3. The mice were kept in an environmentally controlled room (temperature, 25 ± 2 °C; humidity, 60 ± 5%; 12 h light–dark cycle) with free access to food and water.

Mice body weight and food consumption were recorded every week and every another day, respectively. Blood HDL and LDL were monitored biweekly through retroorbital blooding. After being fed with the respective diets for about 12 weeks, the mice were sacrificed. Final body and organ weight were recorded. Mouse blood was collected and serum was separated by centrifugation at 800×g for 15 min at 4 °C. Liver and other organs were removed and snap frozen in liquid N_2_. All samples were stored at −80 °C freezer for further analysis.

### Glucose tolerance tests (GTTs)

A GTTs was performed after mice were fasted for 6 h at the end of the experiment. Tail blood was collected before (0 min) and at 30, 60, 90, and 120 min after administration of a 10.0% D-glucose solution (1.5 g/kg body weight) and blood glucose was measured by an ACCU-CHEK® Active glucometer (Roche Diagnostics GmbH, Mannheim, Germany).

### Biochemical analysis

Serum total triacylglycerol (TG), cholesterol (TC), HDL-C, LDL-C, alanine aminotransferase (ALT), aspartate aminotransferase (AST) of C57BL/6J mice were measured by using Roche P800 chemistry analyzer (Hoffmann-La Roche Ltd., Switzerland) according to the manufacturer’s instructions. The contents of MDA and SOD of serum and liver tissues were measured using commercially available kits (Nanjing Jiancheng Bioengineering Institute, Nanjing, China).

### Histochemical analysis

After mice sacrifice, liver, epididymal fat was excised and weighted. A small piece of liver was fixed in 4.0% formalin solution for 48 h. Then, liver samples were dehydrated, embedded in paraffin wax, sectioned and stained with hematoxylin and eosin (H&E) according to standard procedure.

The morphology of hepatic cell was observed and photographed using a microscope (DM2700P, Leica, Germany). The histological analysis was performed according to the NAFLD scoring system, which was proposed by Kleiner et al. [20].

### Statistical analysis

Data were presented as the mean ± standard deviation (*n* = 10 per group). The statistical analysis was performed by one-way Analysis of Variance (ANOVA) combined with Duncan’s Multiple Range test using SPSS package. *P* < 0.05 was considered significant.

## Results and discussion

### Characteristics of krill oil

By using high quality of krill preserved by a novel procedure with a combination of heat pump drying and freeze-drying process, KO was extracted by a subcritical extraction system with butane [13]. Properties of KO were analyzed and summarized in Tables 1 and 2, respectively. It contained a high amount of polyunsaturated fatty acids (PUFA) specific for omega-3 of DHA and EPA of 16.3% and 9.6%, respectively. DHA and EPA have distinct effect on human health of cardiovascular protection and gain significant increase on demand as nutrient supplements in food industry. KO obtained through this procedure had a very similar fatty acid profiles as reported by others [21] and by Li et al., who used ethanol extraction method [22]. Another feature for KO was its high contents of phospholipids, a component as a nutrient supplement [23]. By using Jiang et al. method [16], the content of the KO was estimated about 62.30% triglycerides (TAG) and 28.68% of phospholipids and high astaxanthin about 248.4 ± 5.2 mg/kg and tocopherols (V_E_) about 67.7 ± 3.2 mg/kg (Table 1), respectively, are other factors for the healthy effect of diet supplement.

**Table 1:** Composition of KO

	Triglycerides (%)	Phospholipids (%)	Astaxanthin (mg/kg)	Tocopherols (mg/kg)	Peroxide value (meq/kg)
Content	62.30 ± 1.2	28.68 ± 1.0	248.4 ± 5.2	67.7 ± 3.2	3.01 ± 0.35

**Table 2:** Fatty acid compositions of KO and lard

Fatty acid	KO (%)	Lard (%)
C12:0	0.2	0.1
C14:0	10.4	1.4
C16:0	21.5	32.8
C16:1	4.0	0.4
C17:0	2.0	0.0
C18:0	1.3	24.6
C18:1	19.4	37.3
C18:2	5.5	3.2
aC18:3	9.2	0.2
C20:0	0.5	0.0
EPA C20:5	16.3	0.0
DHA C22:6	9.6	0.0
∑SFAs	36.2	58.9
∑MUFAs	23.4	40.9
∑*n*-3PUFAs	26.0	0.2

*SFA* saturated fatty acids, *MUFA* monounsaturated fatty acids, *PUFA* polyunsaturated fatty acids

### Effect of KO supplementation on animal health

**Fig. 1:**
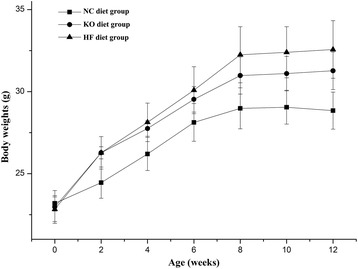
The mice body weight change during feeding

It is known that oil extraction technology such as cold pressed and hot-pressed methods significantly affect the quality and nutrient contents of oils [24]. To further analyze the biological effect of KO produced by our novel procedure [13] on animal health, an animal feeding experiment was performed. High fat diet (HF diet) (20.0% lard based) and with 5.0% substituent of KO (KO diet) were applied to feed C57BL/6 J mice for about 12 weeks (Table 3). As food was freely available to the mice, KO addition in the diet did not affect the amount of food intake (Table 4). Diet is one of critical environmental factors for development of obesity. Increased fat intakes and energy density in diets are associated with body weight gain related to obesity and metabolic diseases [25]. The mice fed with high fat diet increased body weight significantly and continued over the experimental feeding time (Fig. 1). However, the energy consumption did not show significant difference among various different diets groups, since the amounts of food intake for two high fat diets groups (HF diet and KO diet) were decreased about 20.0% compared with chow diet with a low-fat diet (NC diet) (Tables 3 and 4), supporting the notion of energy intensity of diet associated with body weight increase and obesity [26]. Furthermore, the lard based high fat diet contain more saturated fatty acids (Table 2), which could be more prone to obesity and other metabolic disorder such as cardiovascular diseases and hepatic steatosis [27] (as well in this study, see later), had significant more body weight gains and obese than that of NC group. This observation was consistent with high fat animal feeding model and support that balanced diet instead of fat/protein-rich diets are healthier to animals. Interestingly, mice fed with KO containing diet had less potential to increase body weight in comparison with lard based high fat feeding mice (Fig. 1). The body weight gain was probably from obesity as high increase of adipose weight such as epididymal fat (Table 4). HF group had gained about 2.7-fold vs 2.0-fold of KO diet group of epididymal fat weight in comparison to chow group. There was less significant change of other organ weights, such as livers which had about 10% increase for HF diet group and similar for KO diet group compared with the chow diet group. As the only difference between HF and KO diets were 5% substitute of krill oil with lard, these results indicated that components from krill oils could play a benefit role or limit animal body weight gain (see later). This was interesting as KO supplementation in diet could benefit for body weight control in term of current energy rich diets with little restriction on food availability. This result was consistent with others on effect of diets on the animal body weight change [8, 28].

**Table 3:** Composition of experimental diets

Components	NC diet	HF diet	KO diet
Maize starch (g/kg)	654.5	494.5	494.5
Lard oil (g/kg)	0.0	200.0	150.0
KO (g/kg)	0.0	0.0	50.0
Casein (g/kg)	202.9	202.9	202.9
Maltodextrin (g/kg)	50.7	50.7	50.7
Cellulose (g/kg)	50.7	50.7	50.7
DL-Methionine (g/kg)	3.0	3.0	3.0
Sucrose (g/kg)	1.0	1.0	1.0
Choline bitartrate (g/kg)	1.0	1.0	1.0
Sodium chloride (g/kg)	2.0	2.0	2.0
Calcium carbonate (g/kg)	13.2	13.2	13.2
Calcium bicarbonate (g/kg)	10.1	10.1	10.1
Cholesterol (g/kg)	0.0	10.0	10.0
Potassium citrate (g/kg)	10.1	10.1	10.1
Mineral mixture (g/kg)	0.6	0.6	0.6
Vitamin mixture (g/kg)	0.2	0.2	0.2
Energy density (kcal/100 g)	364.4	454.9	454.9

**Table 4:** Food intake and organ weight change of C57BL/6J mice fed for 12 weeks

	NC diet	HF diet	KO diet
Food intake (g animal^−1^·day^−1^)	3.40 ± 0.10^a^	2.65 ± 0.10^b^	2.70 ± 0.10^b^
Liver weight (g)	1.12 ± 0.02^a^	1.25 ± 0.07^b^	1.13 ± 0.09^a^
Epididymal fat (g)	1.50 ± 0.27^a^	4.02 ± 0.46^b^	3.07 ± 1.05^c^

Values were means ± SD (*n* = 10); data that do not share the same superscript letter(s) within a row were significantly different, *p* < 0.05

### KO improved dyslipidemia and liver lipid accumulation

**Fig. 2:**
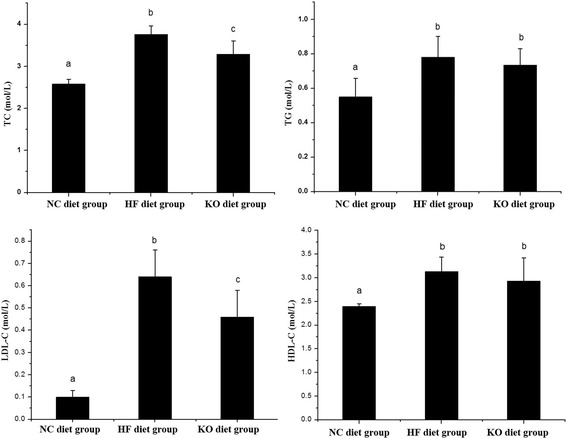
TC, TG, LDL-C, and HDL-C of C57BL/6J mice fed with different diets. Means with different letters (a, b, c) were significantly different from one another by Duncan’s multiple-range test (*P* < 0.05)

The biochemical properties of serum were measured for their change of total triacylglycerol, cholesterol (Fig. 2) and liver functions (Tables 5, 6 and 7). Both total cholesterol and triacylglycerol were reduced after feeding with Krill oil containing diets in comparison with the HF diet. However, the change for total triacylglycerol content was not significant. Further analysis the cholesterol contents of HDL-C and LDL-C in serum showed a reduction of LDL-C and little HDL-C change, indicated that effect of KO on total cholesterol reduction might attribute to the low LDL-C for disposal of cholesterol to the peripheral tissue and lead to improve lipid metabolism in high fat supplement diets.

**Table 5:** Histological characteristics of liver from mice fed different diets for 12 weeks^a^

Item	Definition	Score	Percentage of each category in different groups (*n* = 10 per group)		
			NC diet	HF diet	KO diet
Ballooning	None	0	100	0	80
	Few balloon cells	1	0	10	20
	Many cells/prominent ballooning	2	0	90	0

^a^The histological diagnosis was performed according to the NAFLD scoring systemThe degree of liver cell injury was measured on the point scale 0–2, indicating ballooning in hepatocytes in our present study

**Table 6:** SOD activity and MDA content in serum and liver in C57BL/6J mice

Diets group	Serum		Liver	
	MDA (mmoL/L)	Serum SOD (U/mL)	MDA (mmoL/mg protein)	Liver SOD (U/mLprotein)
NC	21.90 ± 1.38a	137.91 ± 10.39a	1.46 ± 0.01a	28.32 ± 1.29a
HF	28.24 ± 1.33b	114.35 ± 8.48b	1.90 ± 0.02b	24.51 ± 1.01b
KO	22.64 ± 2.13a	155.38 ± 5.32c	1.50 ± 0.03c	29.70 ± 1.34a

Values are means ± SD (*n* = 10); Means with different letters (a, b, c) were significantly different from one another by Duncan’s multiple-range test (*P* < 0.05)

**Table 7:** Serum ALT and AST activities of C57BL/6 Jmice

Diets group	ALT (mmol/L)	AST (mmol/L)
NC	25.82 ± 2.56a	116.54 ± 10.97a
HF	33.84 ± 3.68b	116.86 ± 8.73a
KO	28.66 ± 2.85a	126.25 ± 12.60a

Means with different letters (a, b) were significantly different from one another by Duncan’s multiple-range test (*P* < 0.05)

LDL-C content of the mice was clearly an independent risk factor for atherosclerosis [29] and the rate of LDL-C/HDL-C (Atherosclerosis-index, AI) was a more relevant risk indicator in lipid metabolism disorders [30]. Different diets affected AI with KO diet had better AI than lard based high fat diet, which followed the order: HF diets (0.20) > KO diets (0.15) > NC diets (0.04).

These results of the diets supplement of KO on animal health could decrease the risk of cardiovascular disease, implication on the cardiovascular protective effect of Krill oil which were consistent with other studies. Dietary Krill oil supplementation reduction of hypercholesterolemia has been reported in high fat fed mice [6, 7], rat [28], rabbit [10] and human trial [11, 31].

**Fig. 3:**
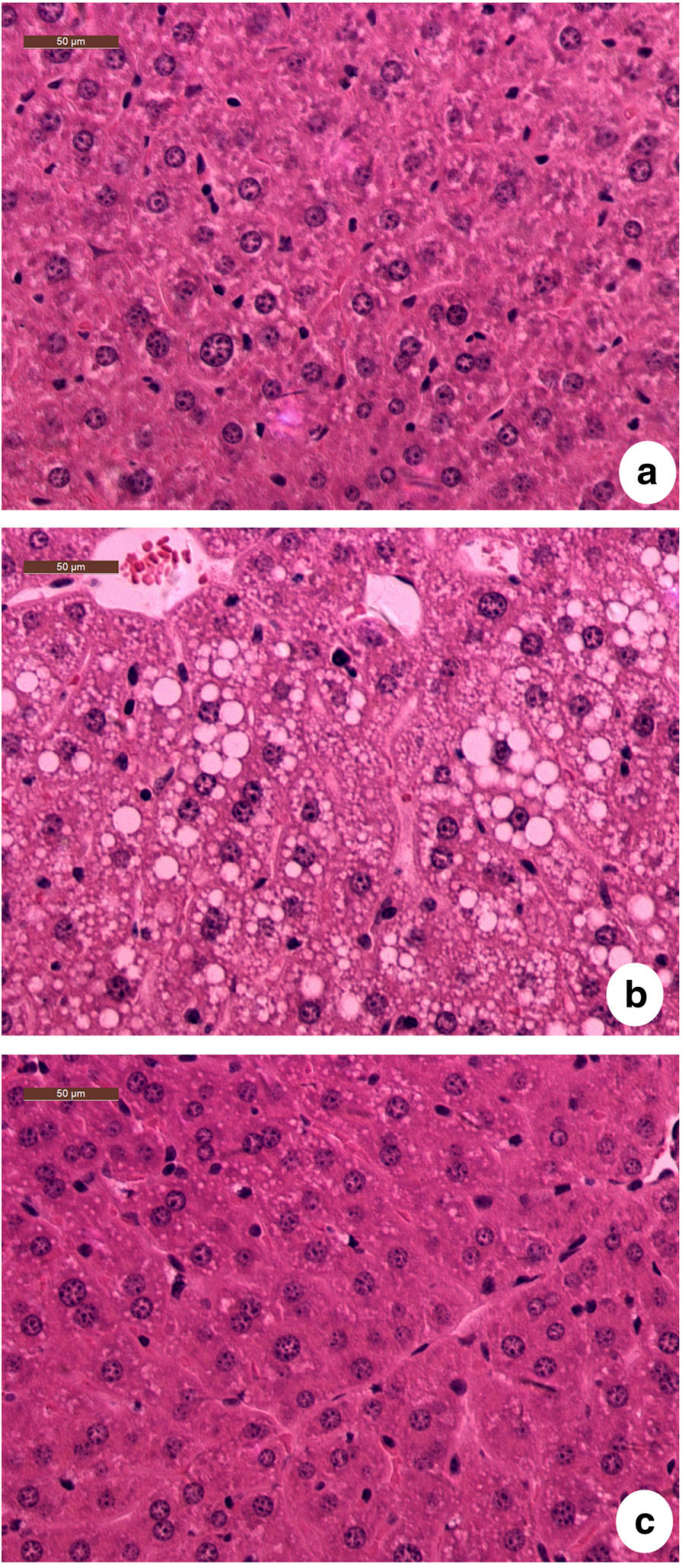
Morphological feature of liver by H&E staining. **a** NC diet group; **b** HF diet group; **c** KO diet group

Lipid accumulation in hepatocytes was observed in the mice fed with high fat diets (Fig. 3). Hepatosteatosis induced by high fat diet was indicated with hepatic tissue staining and categorized with severity of hepatocyte ballooning (Table 5) [20]. The ballooning severity was significantly diminished and liver weights were also reduced (Table 4) with feeding diets containing Krill oil. Krill oil contained a high amount of omega-3 fatty acids of EPA (16.34%) and DHA (9.6%), which are in both of triglyceride and phospholipids. These long chain omega-3 PUFA may attribute its beneficial health effect through alteration of hepatic gene expression, promotion of fatty acid oxidation on lipid metabolism, reduction of inflammation and improvement of insulin sensitivity [32]. Qi et al., reported that omega-3 PUFA containing diets decrease serum triglyceride concentrations in mice by reducing endogenous triglyceride synthesis [33]. KO could inhibit HF diet induced obesity and hepatic triacylglycerol accumulation in mice [34]. Omega-3 PUFA of phospholipids from fish oils suppress hepatic steatosis induced by high fat diet [35]. Compared with fish oil, with quantitatively similar doses of omega-3 PUFAs, KO seems to have a greater potential to promote lipid catabolism [36]. EPA and DHA and its metabolites such as DPA, play an important role in regulation of inflammation. Those Omega-3 fatty acids are also able to mediate anti-inflammatory effect through its metabolites such as resolvins and protectins and insulin-sensitizing effect [37]. In liver, DPA interferes PPARa in the regulation of beta-oxidation and suppression of lipogenic genes. Omega-3 PUFAs involve the suppression of hepatic apoB production and its pool size. DHA unlike other saturated fatty acids such as palmitic acid and oleic acid has less potential to induce ER stress. DPA can inhibit thromboxane synthesis and cause acceleration of the lipoxygenase pathway to affect its biological effect such as inhibition of platelet aggregation [38]. All of these might indicate EPA and DHA beneficial effects are not limited to lipid metabolism as lipid energy resource but may be critical factors on lipid signaling regulation of lipid metabolism and metabolic diseases.

### Effect of KO on glucose metabolism

**Fig. 4:**
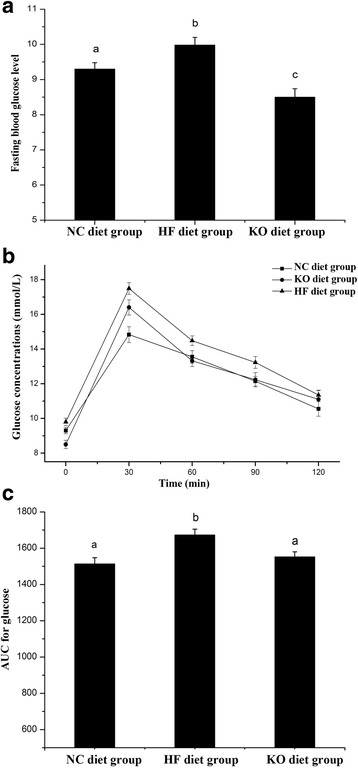
Glucose metabolism change after fed with different diets. **a** Fasting blood glucose level; **b** GTTs for male C57BL/6J mice after 12 weeks on control or different HF diet; **c** AUC for blood glucose over 2 h in GTTs

High fat diets consumption could cause lipid accumulate in the body leading to obesity and other metabolic disorder including glucose metabolism impair and insulin resistance [39]. Feeding with high fat diet, liver insulin resistance could be seen in three days whereas in peripheral tissues it would be about three weeks for insulin resistance [40, 41]. The high fat consumption effect on glucose metabolism was clearly evidenced in this study. As in Fig. 4a, the high fat diet feeding animals led to high fast blood glucose for about 9.8 mmol/L for HF diet in contrast to 9.3 mmol/L of chow diet of NC. With the substituent of 5.0% lard with Krill oil, the fast blood glucose would reduce up to 8.5 mmol/L, which was even more pronounced (10%) than the normal chow group.

Furthermore, effect of Krill oil diets was investigated by using GTTs (Fig. 4c). There was a 13.0% of decrease of AUC of the mice fed with KO containing diet, indicating that supplementation of KO would improve animal glucose metabolism. These results were supported with other studies in animal feeding with Krill oil in rabbit [10], mice [6, 42] and human trial for food supplementation [11], indicating that Krill oil and fish oil supplementation with high content of EPA and DHA indeed could benefit to improving glucose metabolism.

### Effect of KO on oxidative stress

High fat diet feeding to animal significantly induced MDA production, an indicator of lipid peroxidation, and a biomarker of oxidative damage to tissues. In contrast, the SOD activities, capable of removal free radicals in organism, correspondingly, decreased, as in Table 6 for this study. Both in serum and liver tissue, the MDA contents significantly increased with SOD activities diminished. Interestingly, addition of Krill oil in the diet greatly improved and restored the SOD level to that of chow feeding animals. These results likely indicated that there was an oxidative stress in animals particularly in liver due to high fat diet feeding. This was also supported by the increased ALT enzymatic activities in HF group (Table 7). This oxidative stress could be restored by Krill oil feeding might be due to high contents of astaxanthin and tocopherols as well (Table 1). Astaxanthin has high antioxidant capacity which is about ten times greater than β-carotene [43]. Tocopherols are vital for stabilizing the unsaturated fatty acids of the oils against oxidative deterioration [44]. In addition, phospholipids can act as an enhancer for the V_E_ to inhibit the autoxidation [45]. Hence, Krill oil used in this study and prepared from our novel procedure [13], contained abundant of astaxanthin and phospholipids might contribute to their high capacity for ROS removal and antioxidant activities. Krill oil supplementation in diet could also relieve oxidative stress and DNA damages in obese rats [46]. In addition, the metabolites from the high content of EPA and DHA in Krill oil might also contribute to the effect of anti-oxidative and anti-inflammation in the high fat diets feeding animal. The results from this study clearly indicated that the Krill oil used by this study could be good food supplement to animal health.

## Conclusion

The beneficial effect of Krill oil used in this study by a novel procedure was investigated. It was clearly demonstrated that the Krill oil containing a high content of polyunsaturated fatty acids EPA and DHA and astaxanthin could significantly improve dyslipidemia, fatty liver, and glucose metabolism in C57BL/6J mice.

## Abbreviations

ALT: Alanine aminotransferase
AOCS: American Oil Chemists’ Society
AST: Aspartate aminotransferase
AUC: Area under the curve
DHA: Docosahexaenoic acid
DPA: Docosapentenoic acid
EPA: Eicosapentaenoic acid
GTTs: Glucose tolerance tests
H&E: Hematoxylin and eosin
HDL-C: High density lipoprotein- cholesterol
HF: High fat
KO: Antarctic krill oil
LDL-C: Low density lipoprotein- cholesterol
MDA: Malondialdehyde
MUFA: Monounsaturated fatty acids
NC: Normal control
PUFA: Polyunsaturated fatty acids
SFA: Saturated fatty acids
SOD: Superoxide dismutase
TC: Cholesterol
TG: Triacylglycerol
